# Do obese patients with type A aortic dissection benefit from total arch repair through a partial upper sternotomy?

**DOI:** 10.3389/fcvm.2023.1086738

**Published:** 2023-01-27

**Authors:** Lin-Feng Xie, Jian He, Qing-Song Wu, Zhi-Huang Qiu, De-Bin Jiang, Hang-Qi Gao, Liang-wan Chen

**Affiliations:** ^1^Department of Cardiovascular Surgery, Fujian Medical University Union Hospital, Fuzhou, China; ^2^Key Laboratory of Cardio-Thoracic Surgery Fujian Medical University, Fujian Province University, Fuzhou, China; ^3^Fujian Provincial Special Reserve Talents Laboratory, Fujian Medical University Union Hospital, Fuzhou, China

**Keywords:** acute aortic dissection, obesity, partial upper sternotomy, total arch replacement, triple-branched stent

## Abstract

**Background:**

Minimal research has been performed regarding total arch replacement through partial upper sternotomy in patients with acute type A aortic dissection who are obese, and the safety and feasibility of this procedure need to be further investigated. The present study investigated the potential clinical advantages of using a partial upper sternotomy versus a conventional full sternotomy for total arch replacement in patients who were obese.

**Methods:**

This was a retrospective study. From January 2017 to January 2020, a total of 65 acute type A aortic dissection patients who were obese underwent total arch replacement with triple-branched stent graft. Among them, 35 patients underwent traditional full sternotomy, and 30 patients underwent partial upper sternotomy. The perioperative clinical data and postoperative follow-up results of the two groups were collected, and the feasibility and clinical effect of partial upper sternotomy in total arch replacement were summarized.

**Results:**

The in-hospital mortality rates of the two groups were similar. The total operative time, cardiopulmonary bypass, aortic cross-clamp, cerebral perfusion, and deep hypothermic circulatory arrest times were also similar in both groups. The thoracic drainage and postoperative red blood cell transfusion volumes in the partial upper sternotomy group were significantly lower than those in the full sternotomy group. Mechanical ventilation time was shorter in the partial upper sternotomy group than that in the full sternotomy group. Additionally, the incidences of pulmonary infection, hypoxemia, and sternal diaphoresis were lower in the partial upper sternotomy group than those in the full sternotomy group.

**Conclusion:**

This study showed that total arch replacement surgery through a partial upper sternotomy in patients with acute type A aortic dissection who are obese is safe, effective, and superior to full sternotomy in terms of blood loss, postoperative blood transfusion, and respiratory complications.

## 1. Introduction

With recent economic development, living standards have improved significantly. However, the lack of proper exercise and the excessive intake of unhealthy food have led to increased obesity, a condition that can lead to lifestyle and health difficulties. Individuals who are obese often have a combination of hypertension, diabetes, hyperlipidemia, and other risk factors closely related to cardiovascular disease (1, 2). Some studies have reported that obesity is one of the causative factors for the development of type A aortic dissection (AAD) (3). As a result, the proportion of patients with AAD who are obese [Body Mass Index (BMI) ≥30 kg/m2] is continuously increasing. Obesity increases the duration of ventilator use after cardiovascular surgery and can lead to complications such as hypoxemia or poor wound healing, prolonging hospital stays. Since the 1990s, the surgical pathway using partial upper sternotomy (PUS) has been widely used in various cardiac surgeries (4–7). Due to the physiological characteristics of patients who are obese and the urgency of the surgery, such patients have a slow recovery of respiratory function postoperatively and are prone to complications such as pulmonary infection and hypoxemia. Therefore, we believe that total arch replacement (TAR) surgery using PUS has significant benefits for patients with AAD who are obese.

The aim of this study was to investigate whether using PUS is safe and feasible in patients with type A aortic coarctation who are obese and if the potential benefits are associated with the partial incision of PUS versus the full incision required for traditional full sternotomy (FS) in TAR.

## 2. Materials and methods

### 2.1. Patients

This was a retrospective study. Data was collected from the medical charts of patients with AAD treated using TAR between January 2017 and January 2020. Sixty-five patients were categorized into the FS group (35 cases) or the PUS group (30 cases) according to the sternal incision during thoracotomy. All patients met the following criteria: (1) preoperative BMI ≥ 30 kg/m2; (2) acute type A aortic dissection confirmed by aortic computed tomography angiography (CTA); (3) underwent TAR using triple-branched stent graft; and (4) surgery was emergent. The exclusion criteria were: (1) second open-heart surgery; (2) combination of severe trauma or congenital sternal malformation; (3) combination of severe chronic obstructive pulmonary disease (COPD) or severe respiratory insufficiency; (4) need for management of mitral or tricuspid valve lesions during the same period; and (5) need for coronary artery bypass grafting during the same period. Until December 2018, all obese AAAD were treated through the operative approach of FS. Since January 2019, PUS has become the standard operative approach for all obese AAAD. Patients who received PUS did not receive additional preoperative evaluation after excluding the relevant contraindications.

In this study, COPD was defined as chronic bronchitis or emphysema characterized by airflow obstruction and severe respiratory insufficiency was defined as a PaO_2_ < 60 mmHg and a PaCO_2_ > 50 mmHg due to severe disturbance of external respiratory function (8, 9).

All surgeries were performed by the same senior surgeon using the same medical team. The ethics committee of the Union Hospital of Fujian Medical University approved the study. Written informed consent was not required due to the retrospective nature of the study.

### 2.2. Triple-branched stent graft

**Figure 1:**
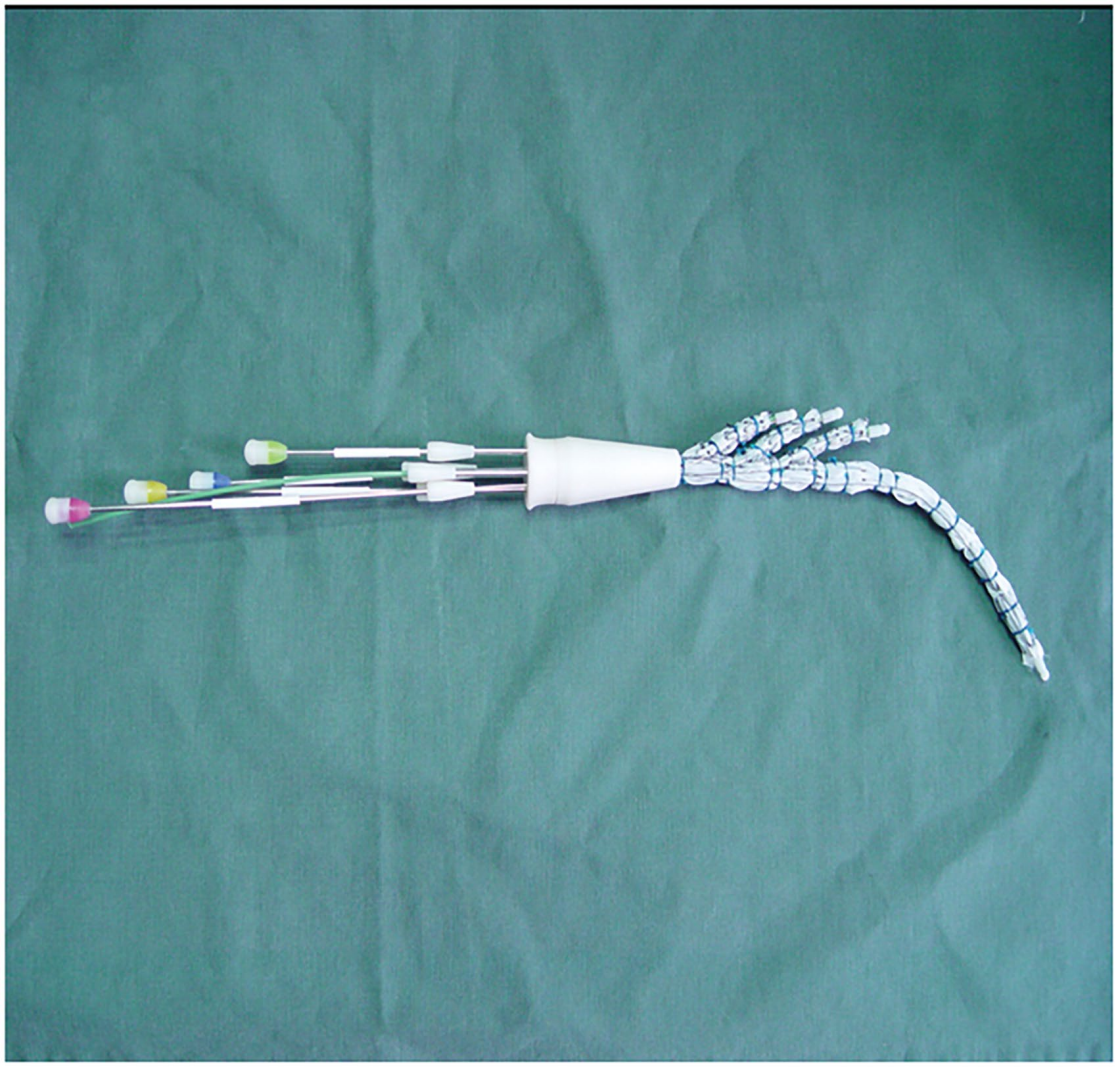
The modified triple-branched stent graft is comprised of a main graft and three sidearm grafts.

**Figure 2:**
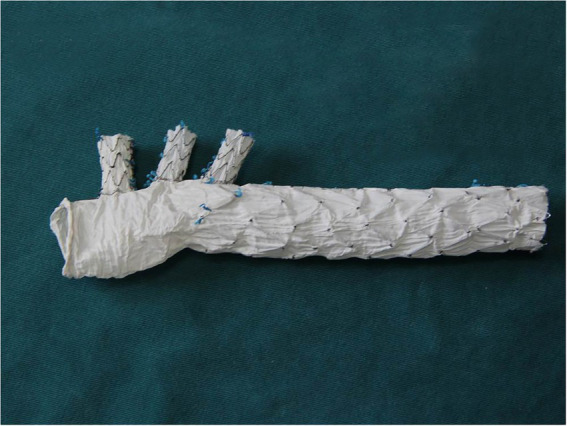
The modified triple-branched stent graft is comprised of a main graft and three sidearm grafts.

The triple-branched stent graft, independently developed by Professor Chen, is a branch-integrated graft composed of a self-expanding nickel-titanium alloy stent and a polyester vascular graft fabric. The triple-branched stent graft includes one main stent and three sidewall stent grafts (Figures 1, 2) (10, 11).

### 2.3. Surgical technique for FS and PUS

**Figure 3:**
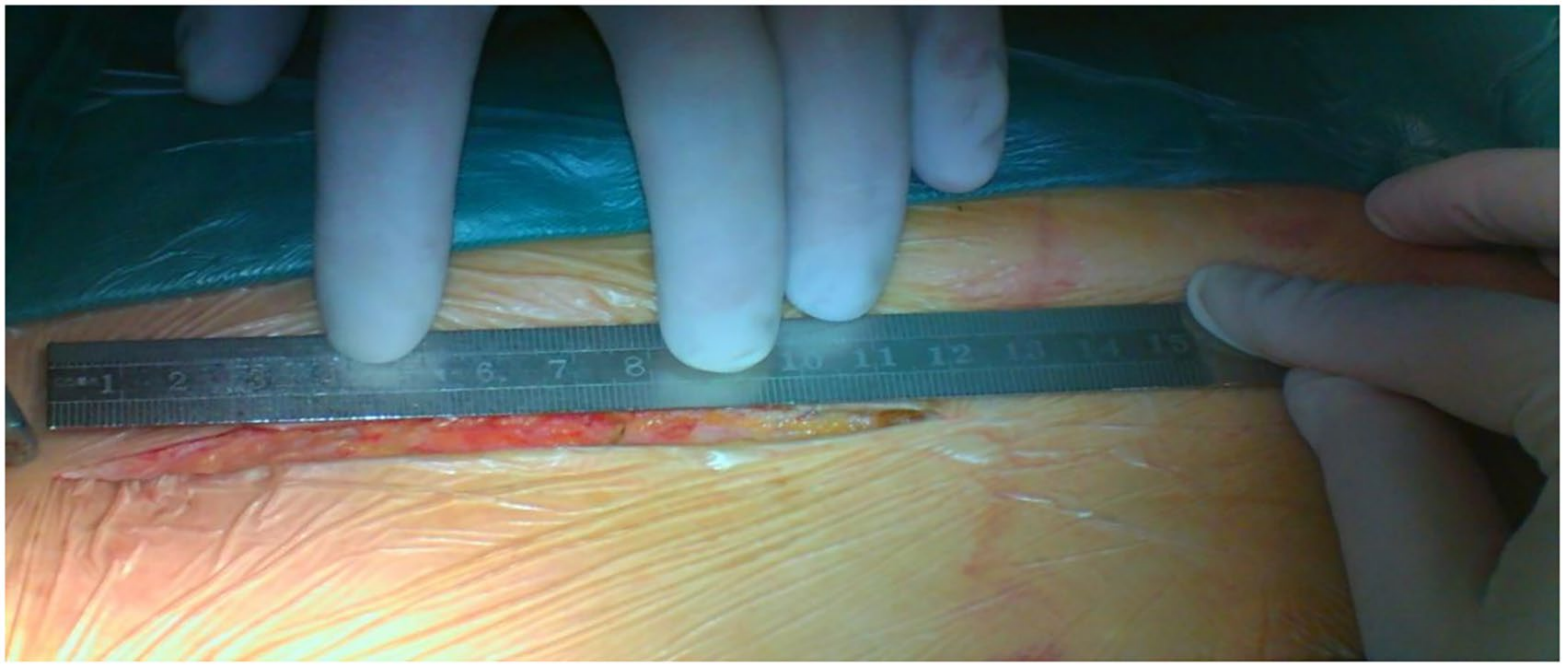
The length of the incision in partial upper sternotomy.

**Figure 4:**
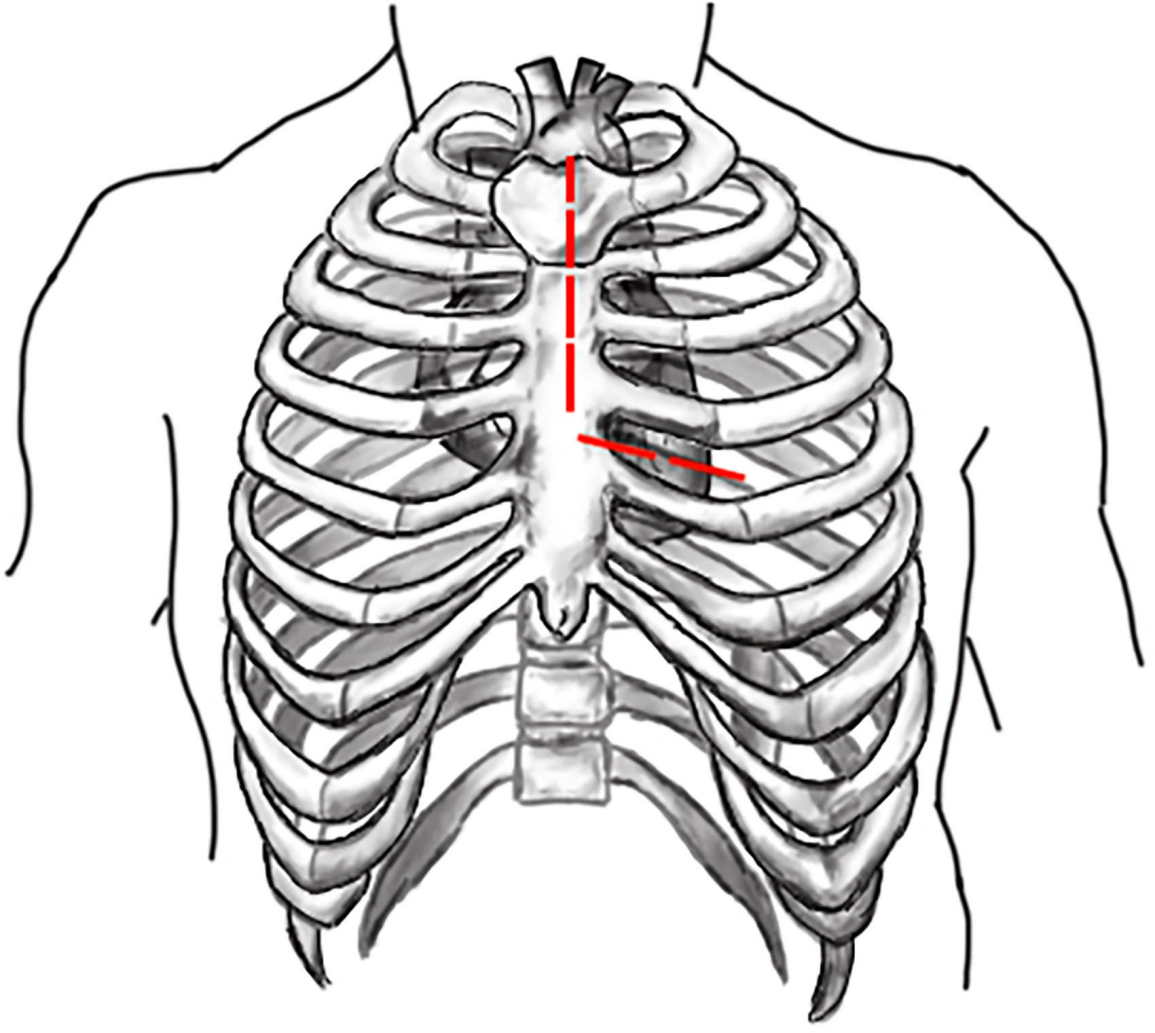
Schematic diagram of “L” shaped incision in partial upper sternotomy.

**Figure 5:**
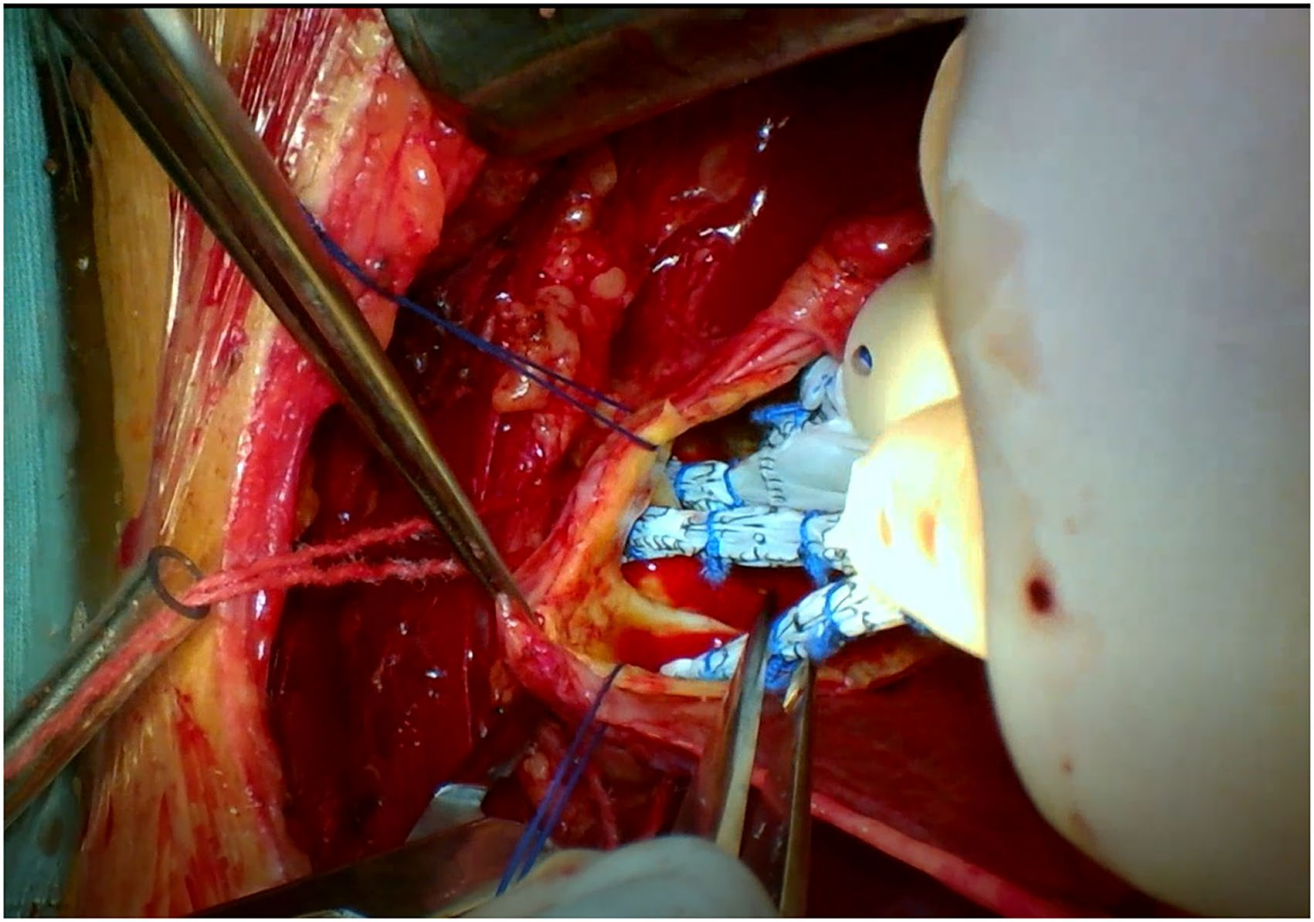
Partial upper sternotomy incision for total arch repair of acute type A aortic dissection.

**Figure 6:**
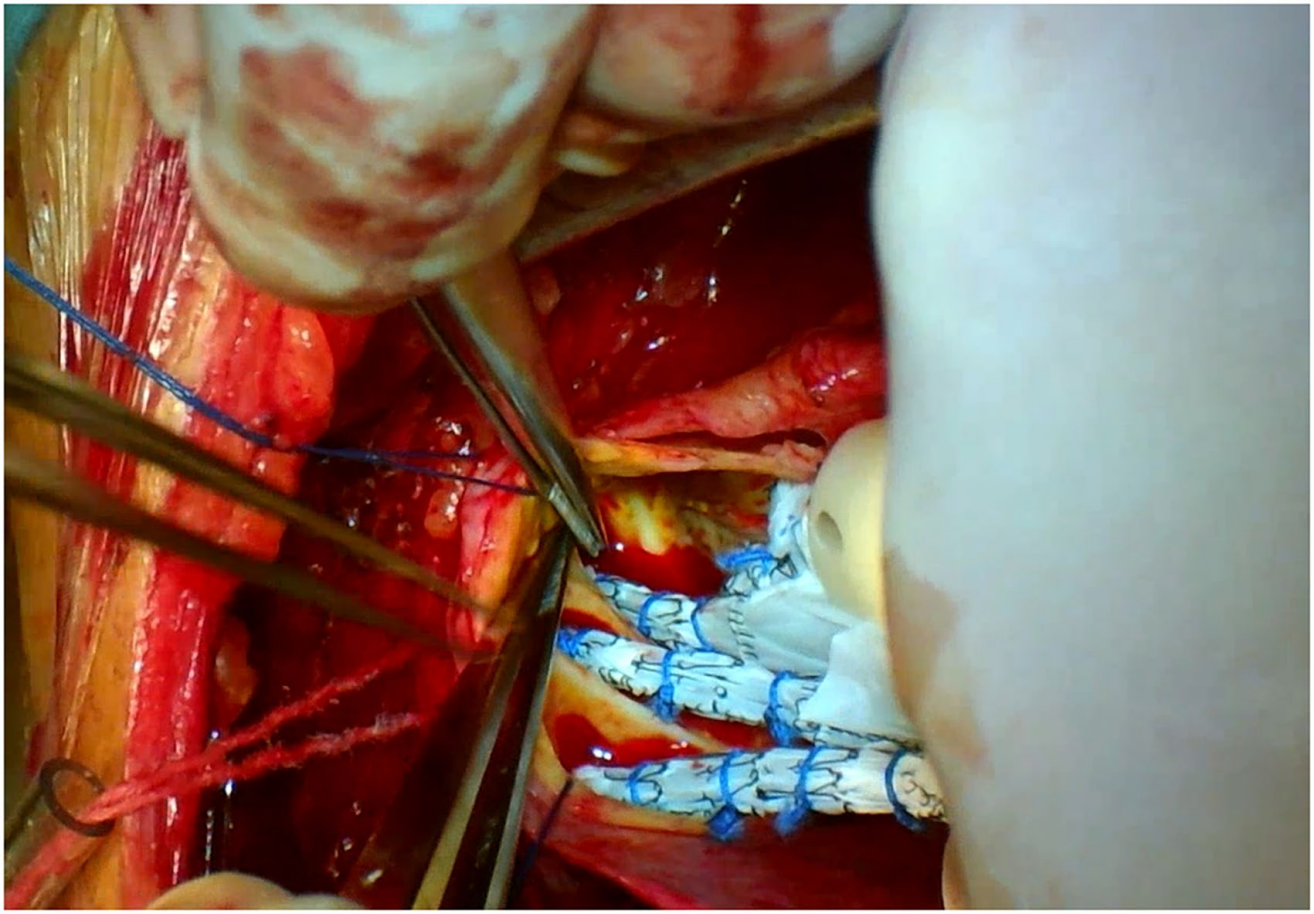
Partial upper sternotomy incision for total arch repair of acute type A aortic dissection.

Both surgeries are performed under general anesthesia. Arterial pressure monitoring of upper and lower extremities is established routinely, and echocardiographic probes are routinely placed in the esophagus. In FS, the skin incision extends longitudinally from the superior sternal fossa to the xiphoid process (a length of approximately 18-20 cm), and the sternum is completely divided. In PUS, the skin incision extends longitudinally from the sternal notch to the fourth intercostal space (ICS) (a length of approximately 8-10 cm; Figure 3). After the surgeon identifies the fourth ICS, the sternum is sawed to the ICS and to the left, creating an “L”-shaped incision (Figure 4). If the incision is not well exposed to the surgical field, it can be extended to the fifth ICS to improve clear exposure. The incision exposes the ascending aorta and the root of the aorta, the superior vena cava, the right atrial appendage, part of the right atrium, and the top of the left atrium. Establishment of systemic cardiopulmonary bypass (CPB) is obtained through a right atrial venous catheter and the femoral and right axillary arteries. After bypass, the body temperature is carefully decreased; when the nasopharyngeal temperature decreases to 32°C, the ascending aorta is blocked above the junction of the sinus canal. The cardioplegia pharmaceutical is directly injected into the coronary vein orifice for cardiac arrest and myocardial protection. If the condition of the aortic root requires attention, the repair is performed using procedures such as aortic sinus plasty and the Bentall. After the aortic root reconstruction is completed, the repaired root is sutured continuously using a pedestrian Dacron vessel. When the nasopharyngeal temperature reaches 25°C, circulatory arrest begins followed by the establishment of unilateral antegrade cerebral perfusion through the right axillary artery cannulation. An oblique incision is made near the small bend of the aortic arch, and the main part of the triple-branched stent graft is inserted into the true cavity of the aortic arch and the proximal descending aorta. Then, each lateral arm graft is located in the aortic branch (Figures 5, 6). Finally, the end of the triple-branched stent graft and the artificial polyester blood vessel are anastomosed continuously to complete the operation (12).

### 2.4. Statistical analysis

We used SPSS version 19.0 for Windows software for all statistical analyzes. Continuous variables with a normal distribution are expressed as mean ± standard deviation (SD) and compared using a Student t-test; otherwise, they are expressed as median (Q25, Q75) and compared with a Mann–Whitney U test. Categorical data were shown as number (%) and analyzed using the chi-square test or Fisher’s exact test as appropriate. *p*-value <0.05 was considered statistically significant. The survival rate was calculated by Kaplan–Meier survival curve, and Log-Rank test was used to test whether there was any difference between groups.

## 3. Results

Between January 2017 and January 2020, 65 patients with AAD who were admitted to our department met the study criteria. Of these, 35 individuals were treated using FS and 30 individuals were treated using PUS. The preoperative baseline information such as age, gender, preoperative BMI, and preoperative cardiac function were similar between the two groups, and the preoperative underlying disease and comorbidities were approximately the same, with no statistically significant differences between the two groups (Table 1).

**Table 1:** Comparison of preoperative data between the two groups (*n* = 65).

Valuables	FS group (*n* = 35)	PUS group (*n* = 30)	*p*-value
Demographic and baseline risks
Age (years)	52.6 ± 5.7	51.7 ± 8.2	0.615
Body mass index (kg/m^2^)	30.7 ± 0.9	30.8 ± 1.0	0.637
Male gender (*n*, %)	24 (68.6%)	19 (63.3%)	0.656
Hypertension (*n*, %)	29 (82.9%)	24 (80.0%)	0.767
Diabetes mellitus (*n*, %)	6 (17.1%)	7 (23.3%)	0.534
Smoking history (*n*, %)	19 (54.3%)	13 (43.3%)	0.379
Drinking history (*n*, %)	7 (20.0%)	4 (13.3%)	0.475
Preoperative LVEF (%)	61.3 ± 9.0	62.0 ± 5.2	0.702
Preoperative complications
Chronic obstructive pulmonary disease (*n*, %)	1 (2.9%)	1 (3.3%)	1.000
Moderate/severe pericardial effusion (*n*, %)	3 (8.6%)	0	0.243
Preoperative renal insufficiency (*n*, %)	4 (11.4%)	2 (6.7%)	0.678
Preoperative hepatic dysfunction (*n*, %)	6 (17.1%)	4 (13.3%)	0.937
Acute aortic regurgitation (*n*, %)	10 (28.6%)	8 (26.7%)	0.864
Pericardial tamponade (*n*, %)	2 (5.7%)	0	0.495
Malperfusion syndrome (*n*, %)	12 (34.3%)	6 (20.0%)	0.199
Transient cerebral ischemia (*n*, %)	1 (2.9%)	0	1.000

Continuous variables were present as mean ± standard deviation (SD). Categorical variables were shown as number (%). The Student t test or Man-Whitney U test was used for continuous variables, and Chi-square test used for categorical variables. LVEF, left ventricular ejection fractions.

No patient required conversion from PUS to FS. The total operative, CPB, aortic cross-clamp (ACC), cerebral perfusion, and deep hypothermic circulatory arrest (DHCA) times were approximately the same in both groups. There were no significant differences in the management of the aortic root between the two groups (Table 2).

**Table 2:** Comparison of intraoperative conditions between the two groups (*n* = 65).

Valuables	FS group (*n* = 35)	PUS group (*n* = 30)	*p*-value
Aortic root procedure			0.965
No treatment (*n*, %)	4 (11.3%)	3 (10.0%)	
Sinus plasty (*n*, %)	20 (57.1%)	19 (63.3%)	
Bentall procedure (*n*, %)	10 (28.6%)	8 (26.7%)	
David procedure (*n*, %)	1 (2.9%)	0	
Intraoperative time
Total operative time (min)	292.1 ± 48.2	283.3 ± 25.5	0.357
Cardiopulmonary bypass time (min)	140.5 ± 19.6	136.0 ± 26.6	0.437
Aortic cross-clamp time (min)	46.2 ± 13.4	44.1 ± 12.8	0.518
Cerebral perfusion time (min)	9.8 ± 2.2	9.3 ± 2.0	0.308
DHCA time (min)	2.0 (2.0,4.0)	2.0 (2.0,3.0)	0.589

Continuous variables were present as mean ± standard deviation (SD) or median (Q25, Q75). Categorical variables were shown as number (%). The Student t test or Man-Whitney U test was used for continuous variables, and Chi-square test used for categorical variables. DHCA, deep hypothermic circulatory arrest.

**Figure 7:**
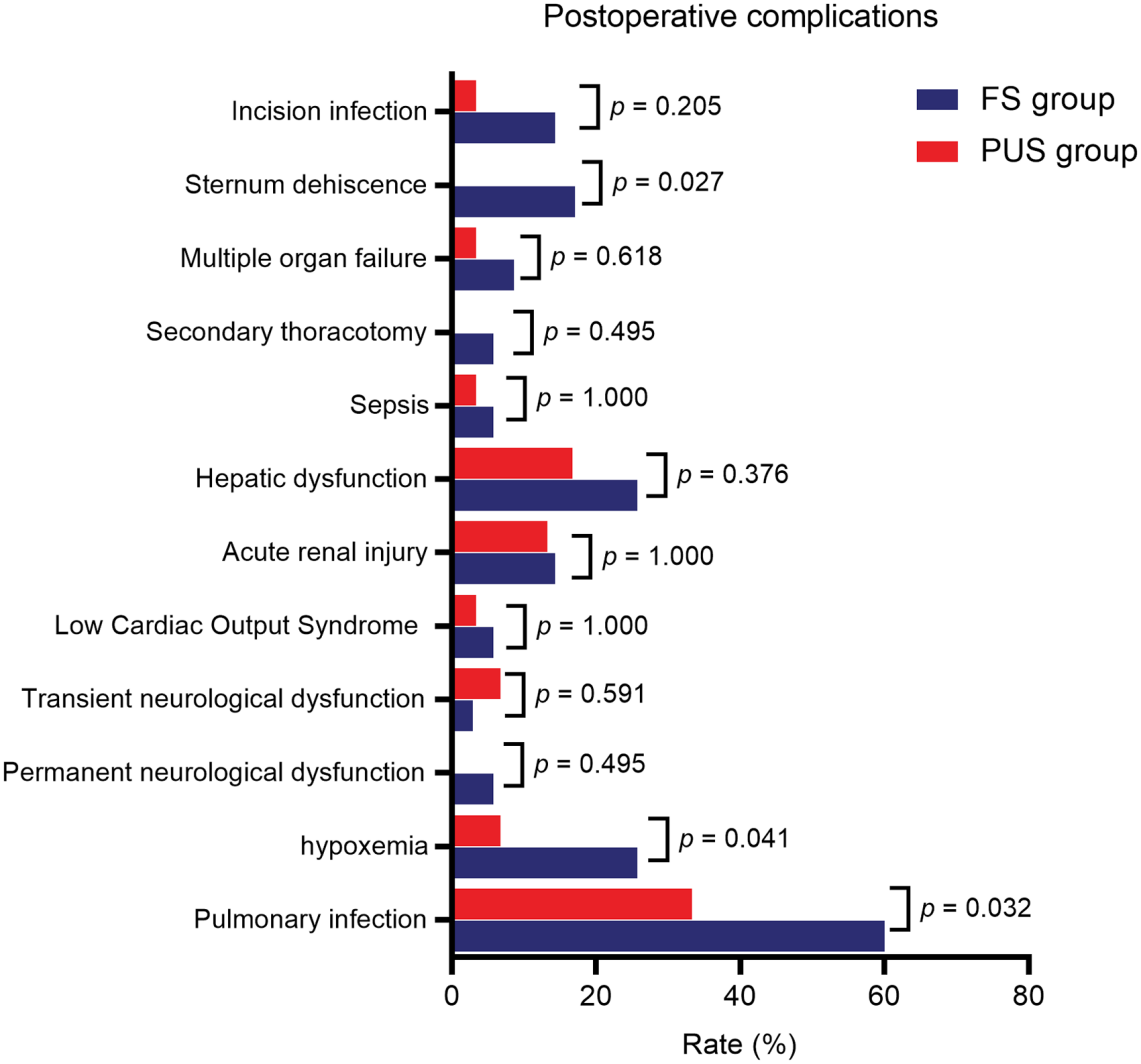
Postoperative complications of patients in two groups.

The postoperative results of the two groups are shown in Table 3. The mean quantity of thoracic drainage volume at 48 h following surgery (572.9 ± 87.6 ml vs. 472.7 ± 115.5 ml, *p* < 0.001) and postoperative red blood cell transfusion volume (697.1 ± 179.4 ml vs. 503.3 ± 145.6 ml, *p* < 0.001) were significantly lower in the PUS group than those in the FS group. The postoperative numeric rating scale pain score was lower [5.5 (5.0, 6.8) vs. 4.0 (3.0, 5.0), *p* < 0.001], and the postoperative mechanical ventilation time was significantly shorter (47.3 ± 13.7 h vs. 40.1 ± 12.7 h, *p* = 0.034) in the PUS group than those in the FS group. The ICU stay time (5.9 ± 5.0d vs. 6.5 ± 4.8d, *p* = 0.652) and postoperative hospital stay time (18.5 ± 5.3d vs. 20.5 ± 7.8d, *p* = 0.223) in the PUS group were shorter than those in the FS group; however, the differences were not significant. Among the postoperative complications, the incidences of pulmonary infection (33.3% vs. 60.0%, *p* = 0.032), hypoxemia (6.7% vs. 25.7%, *p* = 0.041), and sternum refixation (0 vs. 17.1%, *p* = 0.027) were significantly lower in the PUS group than those in the FS group. The incidences of low cardiac output syndrome, hepatic dysfunction, acute renal injury, sepsis, multiple organ failure, and nervous system dysfunction were similar between the two groups. Furthermore, there was no significant difference in the incidence of secondary thoracotomy for hemostasis and surgical wound infection between the two groups (Figure 7).

**Table 3:** Comparison of postoperative results between the two groups (*n* = 65).

Valuables	FS group (*n* = 35)	PUS group (*n* = 30)	*p*-value
30-d mortality (%)	3 (8.6%)	2 (6.7%)	1.000
ICU stay time (day)	6.5 ± 4.8	5.9 ± 5.0	0.652
Total hospital stay time (day)	20.5 ± 7.8	18.5 ± 5.3	0.223
Mechanical ventilation time (h)	47.3 ± 13.7	40.1 ± 12.7	0.034
Thoracic drainage (ml/48 h)	572.9 ± 87.6	472.7 ± 115.5	<0.001
Red blood cell transfusion (ml)	697.1 ± 179.4	503.3 ± 145.6	<0.001
Postoperative NRS score	5.5 (5.0,6.8)	4.0 (3.0,5.0)	<0.001
Postoperative complications
Pulmonary infection (*n*, %)	21 (60.0%)	10 (33.3%)	0.032
Hypoxemia (*n*, %)	9 (25.7%)	2 (6.7%)	0.041
Permanent neurological dysfunction (*n*, %)	2 (5.7%)	0	0.495
Transient neurological dysfunction (*n*, %)	1 (2.9%)	2 (6.7%)	0.591
Low Cardiac Output Syndrome (*n*, %)	2 (5.7%)	1 (3.3%)	1.000
Acute renal injury (*n*, %)	5 (14.3%)	4 (13.3%)	1.000
Hepatic dysfunction (*n*, %)	9 (25.7%)	5 (16.7%)	0.376
Sepsis (*n*, %)	2 (5.7%)	1 (3.3%)	1.000
Secondary thoracotomy (*n*, %)	2 (5.7%)	0	0.495
Multiple organ failure (*n*, %)	3 (8.6%)	1 (3.3%)	0.618
Sternum dehiscence (*n*, %)	6 (17.1%)	0	0.027
Incision infection (*n*, %)	5 (14.3%)	1 (3.3%)	0.205

Continuous variables were present as mean ± standard deviation (SD) or median (Q25, Q75). Categorical variables were shown as number (%). The Student *t* test or Man-Whitney *U* test was used for continuous variables, and Chi-square test used for categorical variables. NRS, numerical rating scale.

**Figure 8:**
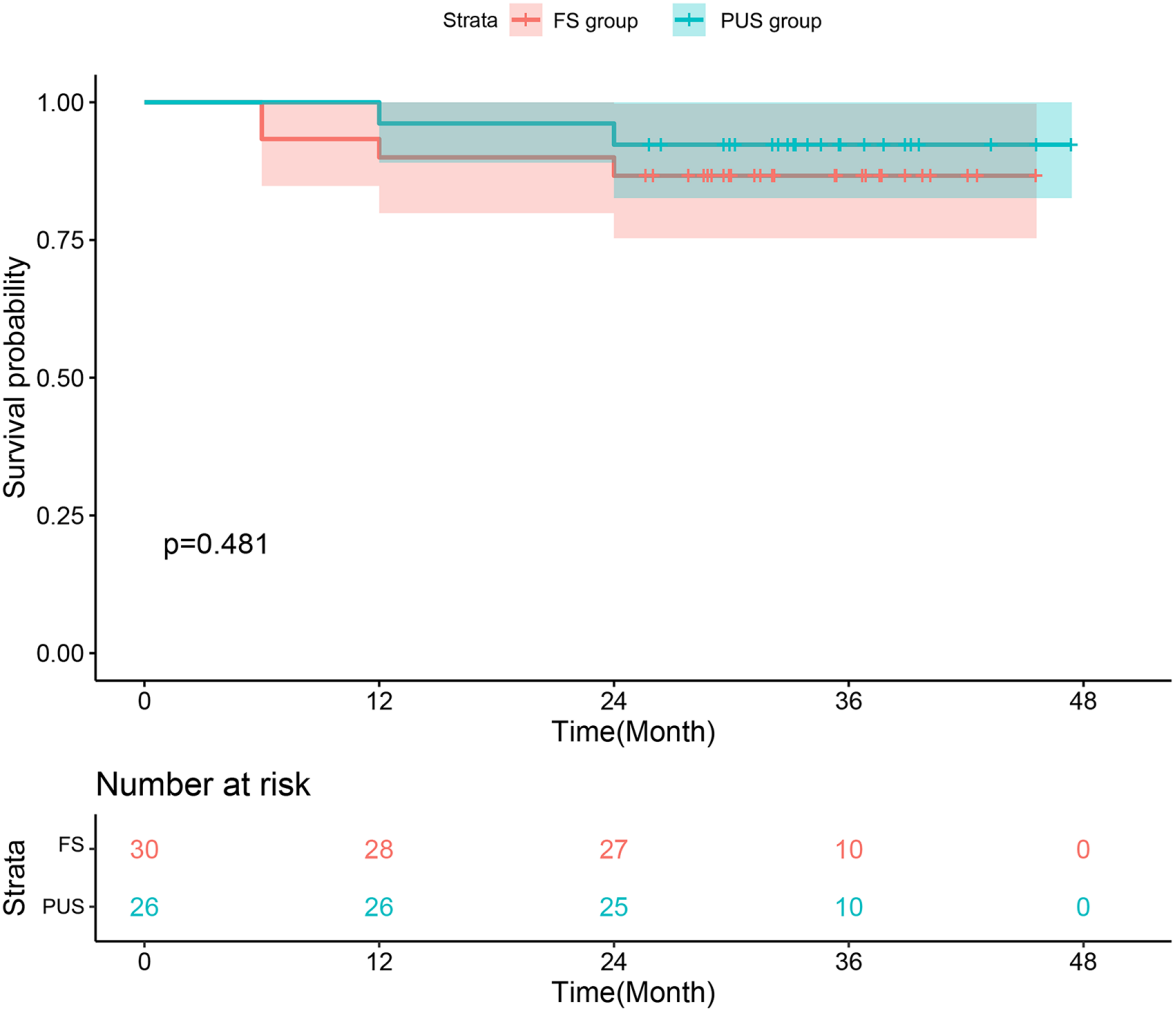
Kaplan–Meier estimates of survival for patients with acute type A aortic dissection who underwent total arch repair with triple-branched stent graft.

The postoperative follow up of the two groups is shown in Table 4. A total of 60 patients were discharged successfully. Up to January 2022, 56 patients were followed up by means of outpatient revisit appointments, telephone, and mail, with a follow-up rate of 93.3% and an average follow-up time of 32.4 ± 8.6 months. The follow-up results of thoracic and abdominal aorta CTA and echocardiography showed no significant difference between the two groups in the closure rate of the false cavity (*p* = 1.000), and the left ventricular ejection fraction was almost the same 2 years after surgery (*p* = 0.230). There was no significant difference between the postoperative survival rate of each time period between the two groups. We combined the survival rate and the postoperative follow-up results to create a cumulative survival function diagram with death as the end point (Figure 8). As Figure 8 shows, there was no significant difference in the early and medium-term survival rate between the two groups (*p* = 0.481).

**Table 4:** Comparison of postoperative follow-up between the two groups.

Valuables	FS group	PUS group	*p*-value
Closure rate of false cavity (2-years)	86.2%	88.5%	1.000
LVEF (2-years) (%)	60.4 ± 4.4	59.0 ± 3.7	0.230
3-month survival rate	100%	100%	1.000
6-month survival rate	93.3%	100%	0.494
1-years survival rate	90.0%	96.2%	0.615
2-years survival rate	86.7%	92.3%	0.675

Continuous variables were present as mean ± standard deviation (SD). Categorical variables were shown as number (%). The Student t test or Man-Whitney *U* test was used for continuous variables, and Chi-square test used for categorical variables. LVEF, left ventricular ejection fractions.

## 4. Discussion

Our results showed that PUS is as safe and effective as FS in patients who are obese. There was no difference in total operative, CPB, ACC, cerebral perfusion, and DHCA times between the two groups. There was also no significant difference in the incidences of secondary thoracotomy for hemostasis, surgical wound infection, low cardiac output syndrome, hepatic dysfunction, acute renal injury, multiple organ failure, and nervous system dysfunction between the two groups. The ICU and hospital stay times were also similar. Most importantly, our results showed that postoperative thoracic drainage and red blood cell transfusion volumes were significantly decreased in the PUS group. Moreover, the incidences of pulmonary infection, hypoxemia, and sternal dehiscence were decreased in the PUS group. Through follow up, we found that the closure rate of the false cavity, the cardiac function, and the early and medium-term survival rates were similar between the two groups.

With the rapid development of minimally invasive surgery, minimally invasive cardiac surgery has become a trend. Since 1996, the partial sternotomy approach has been used in some routine cardiac operations, such as heart valve replacement (13). Recently, PUS has been more widely used in cardiovascular surgeries. In some technologically mature heart centers, PUS has become a routine approach for aortic valve, ascending aorta, and semi-arch replacements (6, 7).

At present, the global population is becoming more and more obese, and the obesity rate continues to rise (14). Due to the large population base and an obesity rate of more than 50%, China has become the largest obese country worldwide (15). The proportion of patients with AAD who are obese also continues to increase. Xie et al. believe that extensive repair of acute AAD using PUS is safe and does not increase the postoperative mortality or risk of serious complications (16). Our results further support this view.

According to Brinkman et al. (17), patients who are obese that have hypertrophy of the chest wall provide challenges for surgeons in terms of achieving adequate surgical field exposure during surgery. Therefore, when using partial sternotomy for cardiovascular surgery in patients who are obese, surgeons will need to be cognizant of the potential issue of inadequate surgical field exposure. Our center independently developed a triple-branched stent graft (10–12). Through the placement of the graft to replace the complex vascular anastomosis in the traditional TAR, the position of the anastomosis of the distal arch vessels in the traditional operation is moved upward to the lesser curved side of the aorta, which is beneficial to the vascular anastomosis operation. This approach also reduces the number of vascular anastomoses, thus avoiding the surgical field exposure problem in patients who are obese.

The sternotomy length is shortened in PUS, reducing the extent of the surgical wound and the risk of postoperative bleeding. Our results showed that postoperative thoracic drainage and red blood cell transfusion volumes were significantly decreased in the PUS group compared to those in the FS group, which is consistent with the advantages of partial sternal incision reported in previous studies (18–21). Due to these advantages, the thoracic drainage tube can be removed earlier in patients who have undergone PUS, which is conducive to early recovery of out-of-bed activities. In addition, the postoperative blood transfusion volume is reduced in PUS; therefore, transfusion-related complications caused by massive blood transfusion are effectively avoided.

The incision in PUS encompasses only part of the upper sternum; the lower sternum remains intact and the overall structure of the thorax is maintained. In patients who are obese, the hypertrophy of the chest wall and the upward shift of the diaphragm increase the pressure in the chest cavity, which in turn causes obstruction of the small pulmonary airways and can lead to difficulty in ventilation and subsequently hypoxia. Furthermore, obesity increases the blood volume in the body, which can easily lead to congestion of lung tissue and decrease lung compliance. Therefore, patients who are obese are prone to difficulty in lung re-expansion in the early postoperative stages. The potential of delayed extubation and the need for longer mechanical ventilation increase the risk of respiratory tract infection. Our results show that PUS can significantly shorten the mechanical ventilation time, significantly reduce the incidences of postoperative pulmonary infection and hypoxemia, and reduce the effect of thoracotomy on respiratory function. This is particularly beneficial for patients who are obese.

Sternal dehiscence is a rare but serious complication after thoracotomy. Patients who are obese have a higher risk of sternal dehiscence due to poor chest compliance and high sternal tension (22). In this study, 6 cases of sternal dehiscence occurred postoperatively in the FS group. In addition, 5 cases in this group were complicated by the comorbidity of type 2 diabetes, and 2 cases had significantly longer mechanical ventilation times postoperatively. These results are consistent with the risk factors of sternal dehiscence reported in the literature (23). There was no sternal dehiscence in the PUS group in our study. This may be largely due to the PUS group having complete sternal structure, reducing excessive tension, thereby avoiding sternal fracture. Hence, mechanical ventilation time was shortened, reducing the effect of positive pressure ventilation on sternal stability and decreasing the probability of sternal dehiscence.

We believe that PUS as a component of FS should be a relatively familiar approach for cardiac surgeons. The only differences between the two approaches are in the mode of thoracotomy and the extent of sternotomy. Therefore, PUS does not significantly change the cardiovascular surgeon’s operating habits. To perform PUS in patients with AAAD who are obese requires the chief surgeon to master all aortic root management methods and be proficient in the use of modified triple-branched stent grafts for TAR. In PUS, the surgical operative space is smaller, which poses more of a challenge to the surgical skills of the chief surgeon and the knowledgeable cooperation of assistant doctors. Therefore, it is necessary for surgeons and assistants to study both procedures extensively and have opportunities for practical experience using PUS in patients who are not obese. This will enhance the success of using PUS in patients who are obese.

This study has some limitations. First, this was a single-center retrospective study. Due to the small sample size, our results may be one-sided. Second, the interval between the operative years (2017–2020) of the two groups is relatively long, and there may have been potential differences in treatment plans and operative techniques. However, this may have been avoided because the same experienced surgeon performed all operations in this study.

### 4.1. Conclusion

Total arch replacement of acute AAD through PUS is a safe and feasible procedure. Compared to TAR through FS, PUS is associated with fewer respiratory complications, better recovery of respiratory function, less blood loss, and improved thoracic stability.

## Data availability statement

The original contributions presented in the study are included in the article/supplementary material, further inquiries can be directed to the corresponding author.

## Ethics statement

The studies involving human participants were reviewed and approved by the ethics committee of the Union Hospital of Fujian Medical University. Written informed consent for participation was not required for this study in accordance with the national legislation and the institutional requirements.

## Author contributions

L-FX, JH, and LC designed the study, participated in the operation, and drafted the manuscript. L-FX and JH collected the clinical data and performed the statistical analysis. LC, Z-HQ, Q-SW, H-QG, and D-BJ provide technical support. All authors have read and approved the final manuscript.

## Funding

This research was sponsored by the National Natural Science Foundation of China (U2005202), Fujian provincial health technology project (2019-ZQN-50), the Natural Science Foundation of Fujian Province (2020 J02056).

## Conflict of interest

The authors declare that the research was conducted in the absence of any commercial or financial relationships that could be construed as a potential conflict of interest.

## Publisher’s note

All claims expressed in this article are solely those of the authors and do not necessarily represent those of their affiliated organizations, or those of the publisher, the editors and the reviewers. Any product that may be evaluated in this article, or claim that may be made by its manufacturer, is not guaranteed or endorsed by the publisher.

## Abbreviations

AAD: type A aortic dissection
PUS: partial upper sternotomy
TAR: total arch replacement
FS: full sternotomy
CPB: cardiopulmonary bypass
ACC: aortic cross-clamp
DHCA: deep hypothermic circulatory arrest

## References

[1] Poirier P Giles TD Bray GA Hong Y Stern JS Pi-Sunyer FX. Obesity and cardiovascular disease: pathophysiology, evaluation, and effect of weight loss: an update of the 1997 American Heart Association scientific statement on obesity and heart disease from the obesity Committee of the Council on nutrition, physical activity, and metabolism. Circulation. (2006) 113:898–918. doi: 10.1161/CIRCULATIONAHA.106.171016 16380542

[2] Jelic S Lederer DJ Adams T Padeletti M Colombo PC Factor PH. Vascular inflammation in obesity and sleep apnea. Circulation. (2010) 121:1014–21. doi: 10.1161/CIRCULATIONAHA.109.900357, PMID: 20159829 PMC2864627

[3] Akutsu K. Etiology of aortic dissection. Gen Thorac Cardiovasc Surg. (2019) 67:271–6. doi: 10.1007/s11748-019-01066-x 30689200

[4] Kocher A Coti I Laufer G Andreas M. Minimally invasive aortic valve replacement through an upper hemisternotomy: the Vienna technique. Eur J Cardiothorac Surg. (2018) 53:ii29–31. doi: 10.1093/ejcts/ezx514, PMID: 29370367

[5] Goebel N Bonte D Salehi-Gilani S Nagib R Ursulescu A Franke UFW. Minimally invasive access aortic arch. Surgery Innovations (Philadelphia, Pa). (2017) 12:351–5. doi: 10.1097/IMI.0000000000000390 28759544

[6] Inoue Y Minatoya K Seike Y Ohmura A Uehara K Sasaki H. Early results of total arch replacement under partial sternotomy. Gen Thorac Cardiovasc Surg. (2018) 66:327–33. doi: 10.1007/s11748-018-0913-2, PMID: 29600320

[7] Deschka H Erler S Machner M El-Ayoubi L Alken A Wimmer-Greinecker G. Surgery of the ascending aorta, root remodelling and aortic arch surgery with circulatory arrest through partial upper sternotomy: results of 50 consecutive cases. Eur J Cardiothorac Surg. (2013) 43:580–4. doi: 10.1093/ejcts/ezs341, PMID: 22700588

[8] Lareau SC Fahy B Meek P Wang A. Chronic obstructive pulmonary disease (COPD). Am J Respir Crit Care Med. (2019) 199:1–2. doi: 10.1164/rccm.1991P1 30592446

[9] Doroshchuk VP. On the definition of the concept of respiratory insufficiency. Ter Arkh. (1965) 37:25–30. 5869665

[10] Chen LW Dai XF Lu L Zhang GC Cao H. Extensive primary repair of the thoracic aorta in acute type a aortic dissection by means of ascending aorta replacement combined with open placement of triple-branched stent graft: early results. Circulation. (2010) 122:1373–8. doi: 10.1161/CIRCULATIONAHA.110.946012, PMID: 20855660

[11] Chen LW Dai XF Zhang GC Lu L. Total aortic arch reconstruction with open placement of triple-branched stent graft for acute type a dissection. J Thorac Cardiovasc Surg. 139:1654–1655.e1. (2010) doi: 10.1016/j.jtcvs.2009.10.022, PMID: 20074749

[12] Chen LW Dai XF Wu XJ Liao DS Hu YN Zhang H. Ascending aorta and Hemiarch replacement combined with modified triple-branched stent graft implantation for repair of acute DeBakey type I aortic dissection. Ann Thorac Surg. (2017) 103:595–601. doi: 10.1016/j.athoracsur.2016.06.017, PMID: 27553503

[13] Cosgrove DM 3rd Sabik JF. Minimally invasive approach for aortic valve operations. Ann Thorac Surg. (1996) 62:596–7. doi: 10.1016/0003-4975(96)00418-3 8694642

[14] Worldwide trends in body-mass index, underweight, overweight, and obesity from. To 2016: a pooled analysis of 2416 population-based measurement studies in 128·9 million children, adolescents, and adults. Lancet (London, England). (1975) 390:2627–42. 10.1016/S0140-6736(17)32129-3 PMC5735219 29029897

[15] Pan XF Wang L Pan A. Epidemiology and determinants of obesity in China. Lancet Diabetes Endocrinol. (2021) 9:373–92. doi: 10.1016/S2213-8587(21)00045-0 34022156

[16] Xie XB Dai XF Fang GH Qiu ZH Jiang DB Chen LW. Extensive repair of acute type a aortic dissection through a partial upper sternotomy and using complete stent-graft replacement of the arch. J Thorac Cardiovasc Surg. (2022) 164:1045–52. doi: 10.1016/j.jtcvs.2020.10.063, PMID: 33223195

[17] Brinkman WT Hoffman W Dewey TM Culica D Prince SL Herbert MA. Aortic valve replacement surgery: comparison of outcomes in matched sternotomy and PORT ACCESS groups. Ann Thorac Surg. (2010) 90:131–5. doi: 10.1016/j.athoracsur.2010.03.055, PMID: 20609763

[18] Mikus E Micari A Calvi S Salomone M Panzavolta M Paris M. Mini-Bentall: an interesting approach for selected patients. Innov (Philadelphia, PA). (2017) 12:41–5. doi: 10.1097/imi.0000000000000337 28129319

[19] Shehada SE Öztürk Ö Wottke M Lange R. Propensity score analysis of outcomes following minimal access versus conventional aortic valve replacement. Eur J Cardiothorac Surg. (2016) 49:464–70. doi: 10.1093/ejcts/ezv061 25732967

[20] Candaele S Herijgers P Demeyere R Flameng W Evers G. Chest pain after partial upper versus complete sternotomy for aortic valve surgery. Acta Cardiol. (2003) 58:17–21. doi: 10.2143/AC.58.1.2005254, PMID: 12625490

[21] Bonacchi M Prifti E Giunti G Frati G Sani G. Does ministernotomy improve postoperative outcome in aortic valve operation? A prospective randomized study. Ann Thorac Surg. (2002) 73:460-5; discussion 465-6. doi: 10.1016/s0003-4975(01)03402-6 11845860

[22] Song DH Lohman RF Renucci JD Jeevanandam V Raman J. Primary sternal plating in high-risk patients prevents mediastinitis. Eur J Cardiothorac Surg. (2004) 26:367–72. doi: 10.1016/j.ejcts.2004.04.038, PMID: 15296898

[23] Gummert JF Barten MJ Hans C Kluge M Doll N Walther T. Mediastinitis and cardiac surgery--an updated risk factor analysis in 10,373 consecutive adult patients. Thorac Cardiovasc Surg. (2002) 50:87–91. doi: 10.1055/s-2002-26691, PMID: 11981708

